# Lithosphere architecture characterized by crust–mantle decoupling controls the formation of orogenic gold deposits

**DOI:** 10.1093/nsr/nwac257

**Published:** 2022-11-16

**Authors:** Zengqian Hou, Qingfei Wang, Haijiang Zhang, Bo Xu, Nian Yu, Rui Wang, David I Groves, Yuanchuan Zheng, Shoucheng Han, Lei Gao, Lin Yang

**Affiliations:** Institute of Geology, Chinese Academy of Geological Sciences, Beijing 100037, China; State Key Laboratory of Geological Processes and Mineral Resources, China University of Geosciences (Beijing), Beijing 100083, China; School of Earth and Space Sciences, University of Science and Technology of China, Hefei 230026, China; State Key Laboratory of Geological Processes and Mineral Resources, China University of Geosciences (Beijing), Beijing 100083, China; School of Electrical Engineering, Chongqing University, Chongqing 400044, China; State Key Laboratory of Geological Processes and Mineral Resources, China University of Geosciences (Beijing), Beijing 100083, China; State Key Laboratory of Geological Processes and Mineral Resources, China University of Geosciences (Beijing), Beijing 100083, China; Centre for Exploration Targeting, University of Western Australia, Crawley 6009, Australia; State Key Laboratory of Geological Processes and Mineral Resources, China University of Geosciences (Beijing), Beijing 100083, China; School of Earth and Space Sciences, University of Science and Technology of China, Hefei 230026, China; School of Earth and Space Sciences, University of Science and Technology of China, Hefei 230026, China; State Key Laboratory of Geological Processes and Mineral Resources, China University of Geosciences (Beijing), Beijing 100083, China

**Keywords:** crust–mantle decoupling, lithosphere architecture, orogenic gold deposit, seismic tomography, magnetotelluric imaging, Tibet

## Abstract

This study, via combined analysis of geophysical and geochemical data, reveals a lithospheric architecture characterized by crust–mantle decoupling and vertical heat-flow conduits that control orogenic gold mineralization in the Ailaoshan gold belt on the southeastern margin of Tibet. The mantle seismic tomography indicates that the crust–mantle decoupled deformation, defined from previous seismic anisotropy analysis, was formed by upwelling and lateral flow of the asthenosphere, driven by deep subduction of the Indian continent. Our magnetotelluric and seismic images show both a vertical conductor across the Moho and high Vp/Vs anomalies both in the uppermost mantle and lowest crust, suggesting that crust–mantle decoupling promotes ponding of mantle-derived basic melts at the base of the crust via a heat-flow conduit. Noble gas isotope and halogen ratios of gold-related ore minerals indicate a mantle source of ore fluid. A rapid decrease in Cl/F ratios of lamprophyres under conditions of 1.2 GPa and 1050°C suggests that the ore fluid was derived from degassing of the basic melts. Similar lithospheric architecture is recognized in other orogenic gold provinces, implying analogous formational controls.

## Teaser

Multidisciplinary research reveals steps of orogenic gold deposits, including mantle partial melting, basic magma ponding and ore fluid release at the base of the crust with decoupled formation from mantle, as well as fluid migration in the middle-upper crust.

## INTRODUCTION

**Figure 1.**
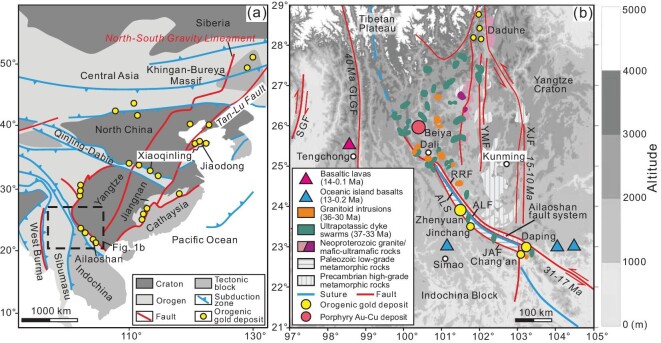
(a) Eastern Asia cratons showing Precambrian blocks (Stanovoy, Khingan-Bureya, North China, Yangtze, Cathaysia) and Phanerozoic orogenic gold provinces at their edges (modified from ref. [4]). (b) Tectonic framework of the eastern Indo-Asian collisional zone (modified from ref. [37]), bounded by Cenozoic faults with ages labeled (ALF: Ailaoshan fault; GLGF: Gaoligong fault; LMT: Longenshan thrust fault; SGF: Sagaing fault; RRF: Red-River fault; XJF: Xiaojiang fault; YMF: Yuanyuan-Muliou fault). Four large orogenic gold deposits (e.g. Zhenyuan, Jinchang, Chang’an and Daping) and the associated Cenozoic igneous rocks along the RRF are shown. A world-class porphyry gold deposit at Beiya and a gold prospect along the Dadu River are shown for comparison.

Orogenic gold deposits (OGDs), which supply ∼30% of our gold resources worldwide, were formed by massive aqueous-carbonic fluid flow along major fault zones during orogeny [1]. Such OGDs formed abundantly in Precambrian greenstone belts during periods of continental growth associated with episodic mantle plumes [2]. Their auriferous ore fluids are traditionally regarded to be derived from dehydration of crustal rocks during prograde metamorphism [1,3–5]. However, there is increasing evidence for the existence of Phanerozoic OGDs that are sited on the margins of Archean cratons (Fig. 1a) [4–9], which usually underwent destruction or reworking due to later oceanic subduction and/or continental collision [9–12] on these margins. These OGDs normally postdated regional prograde metamorphism and were concomitant with mafic dyke swarms [4,13]. Typical examples include the Early Cretaceous OGDs, such as at Apsaka in the Siberian Craton [14] and the Jiaodong Peninsula in the North China Craton [15–17], as well as the Cenozoic OGDs in the Ailaoshan gold belt in the Yangtze Craton [18–21]. Their ore fluids, although contentious [22], were argued to have been derived from a sub-crustal source, with a strong mantle lithosphere affinity [17,23–25]. In view of this, comprehensive studies on the architecture of the lithosphere and geological processes within it are necessary to understand the genesis of such orogenic gold deposits [1,2,11].

The Cenozoic Ailaoshan gold belt, on the southeastern margin of Tibet (Fig. 1b), is an ideal terrane to test and constrain genetic models for such gold deposits. This is because the gold mineralization formed in this belt is anomalously young and well preserved, with the underlying lithosphere most likely to represent that at the time the gold deposits formed. Related previous research includes crustal deformation analysis [26,27], seismic and magnetotelluric (MT) imaging of the lithosphere [28–33], petrological investigation of Cenozoic igneous rocks [34,35], and systematic isotope analyses related to the genesis of OGDs [6]. These data suggest that decoupling of the crust and mantle occurred during deformation, and that low velocity and resistivity anomalies developed at different depths in this region. Although not definitive, the isotopic signatures of sulfur and oxygen for the OGDs imply mantle derivation of the auriferous ore fluid. However, the nature of mantle–crust architecture and its tectonic drivers, especially the nature of the control by lithosphere on the OGDs, was not previously well defined. In this study, we have improved this understanding by reconstructing and refining lithosphere structure utilizing more advanced seismic and MT imaging methods with more densely spaced stations, and by obtaining robust geochemical data, including noble gas (He, Ar, Kr, Xe) and halogen (Cl, F, Br, I) data for gold ores and spatially associated lamprophyres. Our results reveal a lithosphere structure characterized by crust–mantle decoupling with high-flux conduit systems across the Moho, which promoted ponding and degassing of hydrous mantle melts at the mantle–crust transition and controlled the formation of the OGDs.

## GEOLOGICAL BACKGROUND

Ailaoshan, the largest orogenic gold belt in SW China, is located on the southeastern margin of the Tibetan Plateau, which is built on a tectonic collage of continental blocks, including the Sibumasu, Indochina and Yangtze blocks, that amalgamated due to Triassic Paleo-Tethyan and Cenozoic Neo-Tethyan closures [36]. Its tectonic evolution was primarily controlled by the Cenozoic Indo-Asian collision, which resulted in numerous strike-slip faults with block extrusion and Eocene-Miocene magmatism (Fig. 1b).

The Ailaoshan gold belt is controlled by a series of Cenozoic strike-slip faults, including the Red-River fault (RRF), Ailaoshan fault (ALF) and Anding fault (ADF) (Fig. 1b) [37]. This gold belt was developed in juxtaposed high-grade (east) and low-grade (west) metamorphic zones bounded by the ALF. The protolith of the former were largely Neoproterozoic volcanic-sedimentary rocks, recording oceanic subduction beneath the Yangtze Craton [38], whereas the latter is composed of Paleozoic strata and Paleo-Tethyan ophiolitic melanges [39], representing tectonic relics of the Paleo-Tethyan Ocean. The eastern high-grade metamorphic zone experienced amphibolite-granulite facies metamorphism peaking at 44–36 Ma [40,41], followed by near-isothermal decompression at 32–25 Ma and retrograde metamorphism at 25–14 Ma [42]. Due to the India-Asia collision, extrusion of the Indochina block initiated left-lateral shearing along the RRF at 34–30 Ma [40,41,43]. The Eocene ultrapotassic lamprophyre dike swarms (37–28 Ma) with minor granite (used *sensu lato* throughout) stocks (36–30 Ma) intruded at about the same time along the RRF [44,45]. The gold mineralization that is spatially associated with Eocene ultrapotassic magmatism forms numerous large gold deposits, such as the Zhenyuan, Chang’an and Daping deposits (Fig. 1b) [18,20], which are sited in the folded pre-Triassic low-grade metamorphic zone that is bounded by the ADF and ALF (Fig. 1b).

The gold deposits in the Ailaoshan gold belt have features characteristic of OGDs, with two styles of mineralization [18]: quartz vein deposits such as Daping and disseminated ores such as Zhenyuan, Chang’an and Jinchang. Robust radiogenic isotope data define a range of mineralization ages of 45 to 26 Ma [37,46–48], except for the Jinchang deposit with a fuchsite ^40^Ar/^39^Ar age of ca. 60 Ma [49]. Similar timing of gold mineralization is also recorded from the northwestern margin of the Yangtze Craton, where the Dadu River gold prospect with mineralization ages of 30–20 Ma is controlled by the Cenozoic Xiaojiang fault (XJF) system [50]. In addition, the world-class Beiya porphyry gold deposit is spatially and probably genetically associated with Eocene granite stocks and lamprophyre dikes (36–32 Ma) near Dali (Fig. 1b) [51,52].

## RESULTS

### Seismic velocity structure and Vp/Vs model

**Figure 2.**
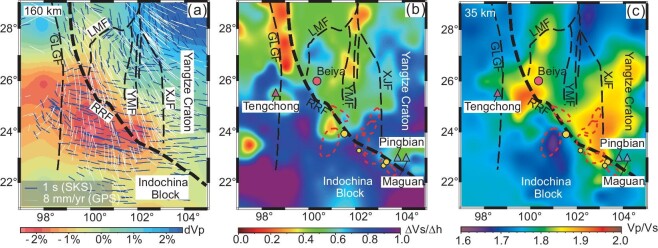
(a) A depth slice of P-velocity image at 160 km. Bounded at ∼26°N, a major low-velocity anomaly at 160 km depth occurs in the southern part with nearly E-W-trending fast directions in mantle (blue line) by SKS shear wave splitting measurements [28,30,72] and nearly N-S-trending surface displacement vectors (white line) by GPS measurements [26,27]. This image shows the lithosphere decoupling deformation induced by the upwelling asthenosphere. (b) Variation in $\Delta {\rm{Vs/}}\Delta {\rm{h}}$ near the Moho. The low-resistivity bodies near the Moho [33] are shown for comparison. For explanations see the text. (c) Variation in Vp/Vs ratios near the actual Moho, reflecting main sites for underplating of basic melts at the crust–mantle boundary interface along the Ailaoshan belt. Abbreviations for fault names are the same as those in Fig. 1.

**Figure 3.**
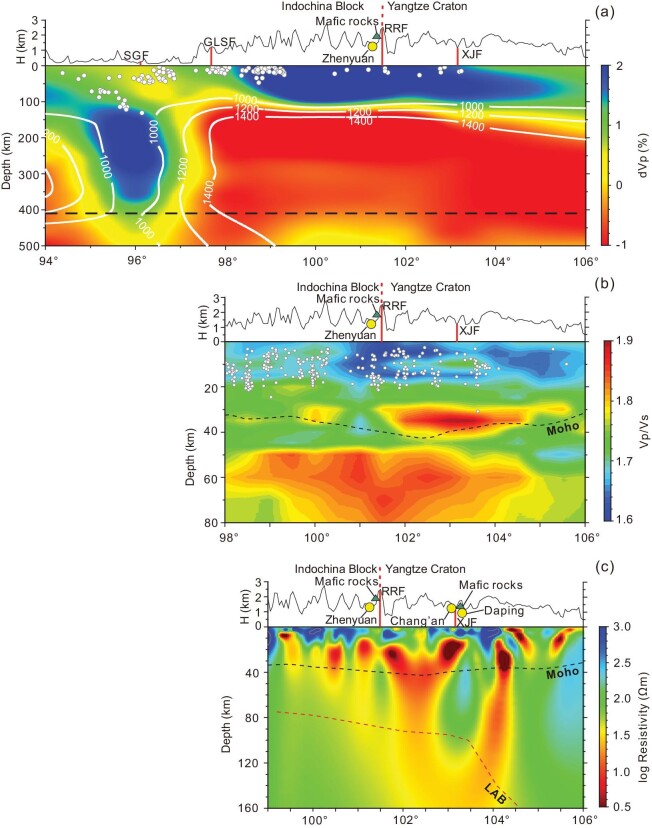
(a) The Vp structures along a profile at 24°N. The 1000°C, 1200°C and 1400°C adiabats of the upper mantle are estimated based on our determination of seismic velocity using the method from ref. [73]. Geological interpretation is given based on seismic velocity features and other geophysical data (see text). (b) Variation in Vp/Vs along a profile at 24°N. Two major domains of high Vp/Vs occur at the Moho (dashed line) and in the uppermost mantle. (c) MT 3D resistivity model projected on a profile near 24°N. Low‐resistivity anomalies beneath the RRF and XJF trend steeply through the Moho and LAB into the asthenosphere. Abbreviation for faults same as Fig. 1b. Mafic dikes (triangles) and the Zhengyuan gold deposit (circles) are shown in (a–c).

A large magnitude low-velocity anomaly is imaged to the south of latitude 26°N at a depth of 160 km by mantle seismic tomography (Fig. 2a). Along the profile at latitude 24°N, this low-velocity anomaly is located on the eastern side of the subducted Indian continent and lies beneath the Asian lithosphere (Indochina and Yangtze) at longitude 97°E–106°E, both of which are imaged as high-velocity anomalies (Fig. 3a). This low-velocity anomaly extends upwards to ∼80 km depth and downwards into the mantle transition zone (Fig. 3a).

The Vp and Vs models from joint inversion of body wave arrival times, surface wave dispersion curves, and receiver functions clearly show a middle-crustal (av 20 km in depth) P-wave and S-wave low-velocity layer (Fig. S1) corresponding to high Vp/Vs values (Fig. S2) and low-resistivity anomalies (Fig. 3c) beneath the Ailaoshan gold belt. The low-velocity layer extends continuously along a profile at 25°N, but gradually declines southward and concentrates at 101°E–103°E beneath the Ailaoshan gold belt (Fig. S1). The S-wave velocity gradients (Vs/h) across the Moho show significant variations in the area. Bounded by the RRF, the Vs gradients in the Indochina block are generally higher than those to the northeast of the RRF. Along the RRF, especially to the north of ∼26°N, there is a low-Vs gradient zone. Similarly, a low-Vs gradient zone also exists along an NS-strike fault system corresponding to the Yuanyuan-Muliou fault (YMF) and XJF and ends at ∼24°N (Fig. 2b).

The Vp/Vs model derived from joint inversion along a profile at 24°N clearly shows a large high Vp/Vs body in the uppermost mantle and two distinct-scale high Vp/Vs anomalies near the Moho (33–40 km) (Fig. 3b). Such a spatially separated high Vp/Vs pattern across the Moho also appears in other profiles across the Ailaoshan gold belt (Fig. S2).

### MT imaging results

A new 3D resistivity model shows that a resistor in the upper crust exists directly beneath the trace of the RRF and XJF, but that some locally spaced low-resistivity anomalies occur in the crust below the gold ore districts (Figs 3c, S3). In particular, there are at least three large low-resistivity anomalies near the Moho at 101–104°E (Fig. 3c). These extend upward into the middle-upper crust and connect to the low-resistivity anomalies below the gold ore deposits. Similar structures are also shown on other profiles from north to south (Fig. S3). Importantly, these low-resistivity anomalies at the Moho penetrate downward across the lithosphere-asthenosphere boundary (LAB) [53], converging into a significant conductor in the upper mantle (Fig. 3c) [33], which is broadly coincident with a high Vp/Vs body (Fig. 3b). Two relatively high-resistivity anomalies in the upper mantle spatially correspond to the Yangtze Craton and Indochina block, respectively (Fig. 3c), reflecting the trans-lithosphere conductors that occur along the boundary of convergent blocks.

### Noble gas and halogen compositions of ore minerals

The He-Ar isotope ratios of disseminated gold ores are equivocal in terms of source as there is fluid-rock interaction during gold mineralization. However, the quartz vein ores, which reflect minimal fluid-rock reactions, are more likely to provide less equivocal indications of fluid source. We therefore analyzed the halogen compositions and noble gas isotope ratios of fluid inclusions hosted in both quartz and auriferous pyrite from samples of quartz vein gold ores hosted by diorite and dolomite at Daping (Supplementary Data, Tables S1–2).

**Figure 4.**
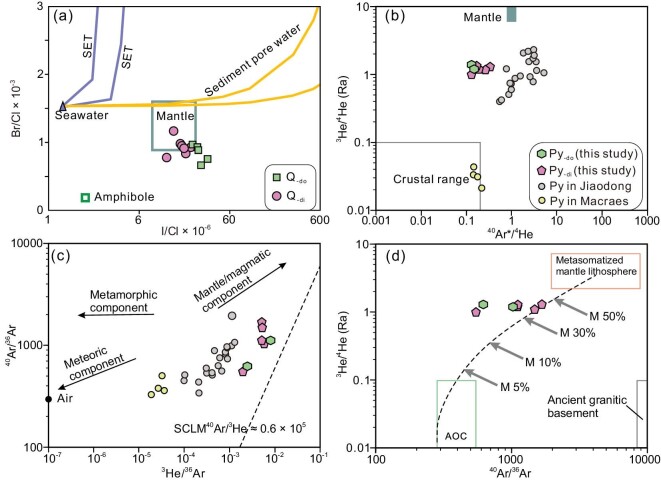
Halogen values and He-Ar isotopic compositions of fluid inclusions from gold-related quartz and pyrite of the Daping gold deposit. (a) I/Cl vs. Br/Cl plot for quartz. (b) ^40^Ar*/^4^He vs. ^3^He/^4^He plot for pyrite. (c) ^3^He/^36^Ar vs. ^40^Ar/^36^Ar plot for pyrite. (d) ^40^Ar/^36^Ar vs. ^3^He/^4^He plot for pyrite and binary mixing modeling for the fluid source. M equals mantle components. The mantle, crust, ancient granitic basement, air, amphibole and sedimentary pore water reference values are from ref. [58] and references therein. The reference values of sediments are from refs [112,113], of mantle halogen ratios from ref. [114], and of seawater from ref. [115] and references therein. The SCLM ^40^Ar/^3^He reference values are from ref. [54]. Abbreviations: Py = pyrite, Py_-di_ = pyrite in diorite, Py_-di_ = pyrite in dolomite, Q_-di_ = ore quartz hosted in diorite, Q_-do_ = ore quartz hosted in dolomite, SCLM = subcontinental lithospheric mantle, SET = seawater trajectories.

Our results show that crushing quartz releases fluids with consistent molar Br/Cl (0.7 × 10^−3^ to 1.2 × 10^−3^) and molar I/Cl ratios (12 × 10^−6^ to 34 × 10^−6^), which are close to mantle values but contrast sharply with the atmosphere endmember (Fig. 4a). Crushing the pyrite samples released 3.7–12.0 × 10^−7^ cm^3^ STP/g ^4^He and 0.7–1.6 × 10^−7^ cm^3^ STP/g ^40^Ar, and yielded a restricted range of ^3^He/^4^He (1.0–1.3 R/Ra) and ^40^Ar/^36^Ar (550 to 1680) ratios (Fig. 4b–d), close to but higher than previous results (0.1 to 1 R/Ra) [54,55]. The fluids trapped in both pyrite and quartz at Daping have consistent ^40^Ar/^36^Ar ratios (Supplementary Data, Tables S1–2), resembling those in the Jiaodong gold deposits with a demonstrated strong mantle contribution [56], but differing from those of Macreas in New Zealand with a proposed predominantly crustal source (Fig. 4b, c) [57]. All samples at Daping have ^3^He/^36^Ar ratios of 2–8 × 10^−3^ (Fig. 4c), similar to mantle values, but higher than those of air-saturated water (5–6 × 10^−8^) [58].

### Volatiles in lamprophyre dikes

**Figure 5.**
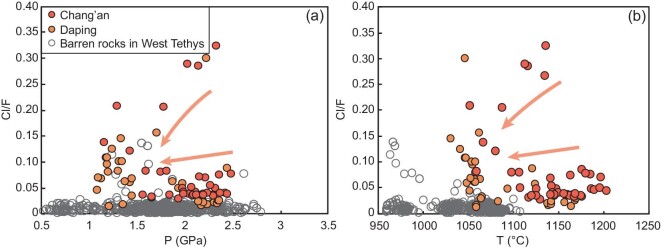
The Cl/F ratios of phlogopites, plus pressure and temperature calculated from phlogopites in lamprophyre dikes. (a) Cl/F vs. P. (b) Cl/F vs. T diagrams. The calculated temperature and pressure of phlogopite is based on refs [116–118]. The detailed calculation process is shown in the Supplementary Data. The Cl/F ratios of phlogopites in lamprophyre first increase and then decrease with a decrease in temperature and pressure.

We analyzed F and Cl contents of phlogopites and estimated pressure and temperature for lamprophyre dikes in the Ailaoshan gold belt (Supplementary Data, Table S3) to better understand relationships between lamprophyres and orogenic gold ores. Our results show that the phlogopites from lamprophyres not spatially associated with gold deposits in western Tethys (Alpine Orogen) have extremely low Cl/F ratios, which has been attributed to the lack of abundant volatiles in their source [59]. In contrast, the lamprophyre dikes spatially associated with both the Chang’an and Daping gold deposits have both demonstrably higher Cl/F ratios and distinctive distributions in terms of pressure-temperature (P-T) conditions compared to those in western Tethys (Fig. 5). The phlogopites in both gold deposits show a similar tendency, in which they first reach maximum Cl/F values at ∼2.2 GPa and 1150°C, and then values rapidly decrease towards 1.2 GPa and 1050°C (Fig. 5).

## DISCUSSION

Our study presents a number of geophysical models, including Vp, Vs, Vs/h and Vp/Vs, as well as resistivity at different scales around the Ailaoshan gold belt (Figs 2, 3). Similar models have been obtained in previous studies for Vp and Vs structure [60–62], Vp/Vs structure [63] and resistivity structure [33,64]. However, Vs/h across the Moho is obtained in the study area for the first time. The application of joint seismic inversion with three different data types helps to improve seismic images of the lithosphere. More densely spaced MT stations in this study also help to provide better electrical images of deep structure. More importantly, the conjunction of these diverse geophysical parameters enables us to reconstruct a more systematic and comprehensive picture of lithospheric structure in order to constrain the deep geodynamic processes that were capable of triggering gold mineralization. The lithospheric structure imaged by seismic and MT methods is the best approximation of the lithospheric structure at the time of mineralization. The mineralization slightly postdated the shearing along the Ailaoshan belt, block rotation of the Indochina block, and extensive magmatic intrusion in the western Yangtze block. Afterwards, the region basically entered a tectonic quiescent stage with crustal uplift and sporadic mafic dike intrusion along the Ailaoshan belt, which does not correspond to the heat flux in both space and intensity.

### Lithospheric deformation and crust–mantle decoupling

The nature of lithosphere deformation and material flow in the southeastern Tibetan Plateau have been constrained using several geophysical imaging methodologies [28–32]. The crustal displacement fields determined by GPS measurements [26,27] indicate that the surface movement involved clockwise rotation around the East Himalayan syntaxis (Fig. 2a), suggesting nearly NS-trending crustal flow across the RRF [28]. However, the pattern of Vs gradients (Vs/h) across the Moho shows that this crustal flow may mainly occur to the north of ∼26°N. Figure 2b displays two nearly NS-trending low-Vs gradient zones along RRF and XJF, north of ∼26°N, corresponding to two previously imaged low-velocity zones [65,66], interpreted to represent two crustal channel flows [67]. In contrast, south of 26°N, the Vs gradients across the Moho have distinct geographically restricted patterns. To the west of the RRF, a large high-Vs gradient domain spatially overlaps with the Indochina block, suggesting that the block is relatively rigid and lacks ductile crustal rheology and Moho reworking. To the east of the RRF, a low-Vs gradient zone, bounded by the Xiaojiang and Yuanyuan-Muliou faults, is truncated by the RRF at 24°N (Fig. 2b). Such an abrupt change suggests that crustal flow was likely blocked by the large-scale strike-slip RRF starting at ∼26°N, where the Indochina block is interpreted to have been extruded along the RRF.

Many studies of seismic anisotropy in the southeastern Tibetan Plateau show that the polarization directions in the crust are dominantly N-S [67–70], consistent with geodetic estimates of crustal displacement (Fig. 2a) and the N-S-trending rock fabrics interpreted to be caused by crustal compression and shearing. In contrast, the mantle anisotropy derived from analysis of teleseismic shear wave splitting has a pronounced transition in polarization direction from primarily N-S to mostly E-W around latitude 26°N (Fig. 2a) [28–30,62]. This transition reflects a fundamental change in the deformation regime within the southeastern margin of Tibet along the RRF. Recent seismic anisotropy studies have ascribed the E-W orientation of seismic anisotropy to deformation from the upper asthenosphere to the mantle lithosphere [32,62,71].

North of ∼26°N, the anisotropy can be interpreted to represent coherent deformation between the crust and mantle lithosphere [28,30,72]. South of ∼26°N, however, the SKS polarization directions do not correlate with known surface features and GPS measured data (Fig. 2a), implying that deformation between crust and mantle was mechanically decoupled [29]. It is noteworthy that this large-scale domain of decoupled deformation spatially overlaps with the extensive low-velocity body in the upper mantle (Fig. 2a). This decoupled deformation between crust and mantle constitutes a specialized architecture for the Ailaoshan gold belt.

### Asthenosphere driving lithospheric crust–mantle decoupling

Our seismic Vp image demonstrates strong velocity heterogeneity in the upper mantle (Figs 2a, 3a). The profile at 24°N clearly depicts that the Indian lithosphere, as a high-Vp body, experienced high-angle subduction and reached the mantle transition zone (Fig. 3a). To the east of the subducted Indian slab, a large-magnitude mushroom-shaped low-Vp body extends laterally beneath the rigid Asian mantle lithosphere, with its stem rooted into the deep mantle (Fig. 3a). We interpret this effect as representing upwelling asthenosphere driven by eastward deep subduction of the Indian lithosphere. Our seismic images depict that the upper interface of the asthenosphere is at a depth of ∼80 km (Fig. 3a), consistent with the LAB depth (80–100 km) beneath the Ailaoshan gold belt [53]. These data are consistent with extensive thinning of the overlying Asian lithosphere caused by upwelling asthenosphere.

Figure 3a denotes the adiabat of the upper mantle calculated using a velocity-temperature model [73], which defines a steep temperature gradient centered on the upwelling asthenosphere. We estimate that the mantle temperatures at ∼80 km were up to 1000°C (Fig. 3a), consistent with those recorded by mantle xenoliths at Maguan (928–1110°C at 50–80 km [74]). This estimated temperature is also close to the estimated equilibrium temperatures (1007–1130°C) for the Quaternary basalts at Tengchong [75] and those (1000–1150°C) of Eocene lamprophyres along the RRF [76]. In addition, this mantle temperature approximates the solidus temperature of the H_2_O-bearing mantle peridotite at similar pressures [77]. These data suggest that the upwelling asthenosphere provided enough heat for partial melting of the overlying Asian mantle lithosphere since the Eocene. At a deeper level of 82–120 km, where ocean-island basalts (OIBs) originate [78], the calculated mantle temperatures reach the lowest melting temperature (1200°C) of the mantle peridotite [77], but lower than the estimated equilibrium temperature (<1373°C) of the OIB magmas at Maguan [79]. This means that melt segregation from the limited partially melted upwelling asthenosphere could have led to the generation of OIB magmas.

**Figure 6.**
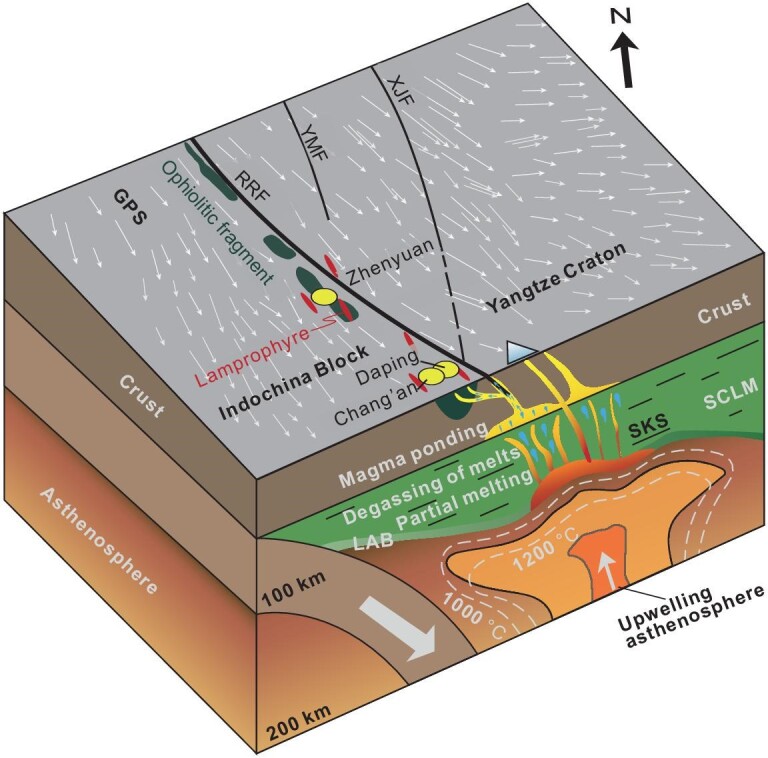
A cartoon illustrating a lithosphere architecture characterized by crust–mantle decoupling, constrained by geophysical and geological data, and its control over generation of orogenic gold deposits. The subduction of the Indian continent induced E-W-trending flow of mantle lithosphere driven by asthenosphere upwelling and NNW-trending crustal deformation, constituting an architecture characterized by crust–mantle decoupling. Controlled by this architecture, the RRF acted as a high-flux conduit, along which basic magma ponded and degassed, leading to formation of a series of OGDs at the fault intersections. Abbreviations: LAB = lithosphere-asthenosphere boundary; SCLM = subcontinental lithospheric mantle.

The above P-T estimates for the mantle lithosphere, combined with the temporal-spatial distribution of the Cenozoic magmatic rocks south of 26°N (Fig. 1b), further constrain a sub-lithosphere process beneath the Ailaoshan gold belt. This process is interpreted to include vertical upwelling of the asthenosphere that triggered mantle melting focused at 35 Ma, lateral flow that produced the Tengchong basalts at 14 Ma, and continued upwelling and melt segregation that generated E-W-trending sporadic OIB magmatic rocks since 13 Ma. We therefore suggest that vertical upwelling and lateral flow of the asthenosphere, driven by eastward deep subduction of the Indian continent during Cenozoic collision, caused E-W-directed deformation and extension of the overlying Asian mantle lithosphere (Fig. 6).

### Heat-flow conduits and ponding of basic magma

The emplacement of the Eocene lamprophyre dikes along the RRF (Fig. 1b) suggests that this block boundary fault likely provided a channel for heat flow from the upper mantle (Fig. 6), which is supported by low-Vs gradients across the reworked Moho by upwelling heat flow along the RRF (Fig. 2b). Seismic receiver functions analysis indicates that the converted phases at both Moho and LAB beneath the RRF show significant lateral discontinuities, suggesting a lithosphere-scale vertical discontinuity and mechanically weak zone [33]. The heat-flow data suggest that heat flow via this weak zone strongly modified the crustal thermal state beneath the RRF. For example, the lowermost crust at Dali, near the RRF, has an abnormally high geothermal temperature estimated as being up to 1000°C [80], thus triggering its melting to generate the Eocene gold-rich porphyries at Beiya [52]. The heat flow along the RRF also resulted in high-temperature granulite-facies metamorphism of the lower crust at 40–35 Ma [42].

Our MT model further constrains the heat-flow conduit across the Moho. It shows that the spherical conductors near the Moho extend downwards and converge into a large conductor in the upper mantle (Figs 3c, S3). This remarkable low-resistivity feature cannot easily be explained by the presence of relatively dry upper mantle, based on anhydrous mantle xenoliths [81], requiring the involvement of melt and/or water. The maximum melt fraction is up to 3% in the uppermost mantle, based on the analysis of mantle H^+^ water and melt content [33]. This requires that the heat-flow conduits were rooted in the underlying mantle lithosphere containing a small amount of melt and traversed across the Moho along the Ailaoshan belt.

This interpretation is also consistent with our Vp/Vs model, which shows two spatially separated high Vp/Vs anomalies at 30–40 km and 60–80 km beneath the RRF (Fig. 3b). Theoretically, such high Vp/Vs ratios could be largely attributed to more mafic rocks and/or partial melts [82]. The high Vp/Vs body in the uppermost mantle (60–80 km) is rooted in the underlying asthenosphere as shown by the high-magnitude low-velocity anomaly (Fig. 3a, b), suggesting that the partially melted mantle lithosphere was heated by upwelling asthenosphere. High Vp/Vs anomalies at the base of the crustal base spatially coincide with the lower-crustal conductors that extend upward into gold ore districts (Fig. 3b, c, Fig. S4), consistent with a model in which hydrous basic to ultrabasic magmas ponded largely at the base of the crust, but partly intruded into the upper crust as lamprophyre dikes.

Integrated analysis of the Vp structure, Vp/Vs ratio and the MT model combined with geological observations indicates that ponding of mantle-derived basic magmas at the base of the crust was largely concentrated at two locations along the RRF. The first is located east of the RRF, where the OGDs spatially occupy the edges of a Vp/Vs anomaly at a depth of 35 km (Fig. 2c). For example, the Zhenyuan gold district is located at an intersection between the RRF and YMF at ∼24°N (Fig. 2c), where the crustal conductors coincide with a high Vp/Vs body in the lower crust (Fig. 3b, c), and Vs/h reflects strong velocity modification near the Moho (Fig. 2b). The Chang’an and Daping gold districts are located at an intersection of the RRF with the XJF at ∼23°N, where the high Vp/Vs mantle anomalies are spatially coincident with high mantle conductors beneath the gold districts (Fig. S4), and variation in Vs/h suggests a local thermal disturbance at the Moho (Fig. 2b). All these data lead us to conclude that the block boundary at the RRF provided channels for high heat flow from the partially melted mantle lithosphere, and that decoupling between crust and mantle deformation promoted ponding of basic magmas at the base of the crust with limited intrusion into the upper crust. The second ponding location is around Dali, where basic magmas ascended via heat-flow conduits and ponded at the base of the crust, leading to partial melting of the lower crust to generate hydrous magmas that were the parents of the porphyry-gold deposit at Beiya (Fig. 2c). We therefore argue that the lithospheric architecture that was characterized by crust–mantle decoupling and heat-flow conduits across the Moho had a profound impact on the formation of the Ailaoshan gold province.

### Link between basic magmas and gold deposits

Phlogopites, as the largest and most abundant phenocrysts in lamprophyres, are able to dynamically re-equilibrate their composition in the melts [83], and thus can preserve a halogen record representative of the melt composition during crystallization [84]. The halogens, especially Cl and F, have been recently applied to trace and evaluate the role of magmatic volatiles, according to their sensitivity to volatile degassing [85] and their similar properties to incompatible elements [86]. The concentrations of halogens in a magma would gradually increase with its evolution and, accordingly, Cl/F ratios would increase due to the greater partition coefficient of Cl than F in volatile-rich melts such as those crystallizing lamprophyres. However, the exsolution of fluid by degassing would remove a large amount of Cl from the melt into the fluid, leading to a significant decrease in Cl/F ratios in the evolved magmas [87].

The Cl/F ratios of phlogopites from lamprophyres in the Ailaoshan gold belt continuously decrease, with P and T ranging from 2.4 GPa to 1.2 GPa and 1150°C to 1050°C (Fig. 5). Cl/F ratios demonstrate a particularly rapid decrease at 1.2 GPa and 1050°C (Fig. 5), which corresponds to the depth of the crust–mantle boundary (35–40 km; Fig. 3) and the high geothermal temperature of up to 1000°C at the Moho [80]. This indicates that a large amount of Cl was extracted by degassing of the hydrous magmas at the interface of the crust–mantle transition. This degassing process is also recorded by some special textures of the lamprophyre dikes, such as gas cavities and abundant carbonate ocelli [34].

The noble gas and halogen contents and ratios of fluids trapped in ore minerals can constrain whether the fluid that exsolved from the basic magma has the capacity to generate gold ores, since the noble gases and halogens are normally stable during fluid transport [57,88] and therefore can effectively distinguish between different fluid reservoirs [89]. Previous He-Ar isotope analyses on fluid inclusions from gold-related pyrites yielded a range of ^3^He/^4^He of 0.1 to 1 R/Ra [54,90], suggesting a mantle contribution to the ore fluid [90]. Our new data show relatively high ^3^He/^4^He ratios (1.0 to 1.3 R/Ra) and ^3^He/^36^Ar ratios (2–8 × 10^−3^), confirming a significant contribution from mantle or degassing mantle melts to the ore fluid. In particular, the fluid inclusions from minerals in quartz vein orebodies have molar Br/Cl ratios of 0.7–1.2 × 10^−3^and I/Cl ratios of 12–34 × 10^−6^, identical to those of mantle values (Fig. 4a), further demonstrating that ore fluids had the potential to be formed by degassing of hydrous basic magma at the base of the crust.

The conclusions obtained via new geochemical data are supported by Pb-S isotope data. The lamprophyre dikes have narrow ranges of ^206^Pb/^204^Pb (18.50–18.59), ^207^Pb/^204^Pb (15.60–15.65) and ^208^Pb/^204^Pb (38.75–38.84), which overlap with those of gold-related sulfides in the Ailaoshan gold belt (Supplementary Data) [91,92]. The gold-rich rims of ore pyrites uniformly display a limited range of δ^34^S values around 0‰ [6,93,94], similar to those expected for a mantle source for the lamprophyre dikes that are spatially related to the gold deposits.

The lamprophyre dikes themselves have too low a volume to directly exsolve ore fluid to form the gold deposits but provide a link to related but larger-volume subcrustal magma-related devolatilization processes that do have this capacity. Thus, the spatially and temporally related gold deposits and lamprophyres do not have a direct genetic relationship but are indirectly connected to a similar mantle lithosphere source and belong to the different consequences of the same mantle process. The platinum group element (PGE) analyses carried out in a previous study have shown that the basic magma has witnessed sulfide saturation and separation [6,95], which would increase the fertility of the fluid source.

### Crustal conduits for ore fluid migration

Our MT imaging reveals vertical or oblique high-strength conductors in the middle-upper crust (Figs 3c, S4). For example, a high-strength conductor that is inclined to the NE extends obliquely downward to 40 km beneath the Zhenyuan ore district (Fig. 3c). Similarly, two high-strength conductors beneath the Chang’an and Daping ore districts intersect near the Moho (Fig. S3). These conductors cannot be explained by the widely exposed high-grade metamorphic rocks in the Ailaoshan belt, which typically display low conductivity [33]. A possible interpretation is that the ophiolitic fragments or oceanic relics, which survived underneath the Ailaoshan belt [96] and were strongly replaced by heat fluid flows, migrated along the faults. This is similar to the MT structures, called Fingers of God, from below the supergiant Olympic Dam iron-oxide copper-gold deposit, which are interpreted to represent fluid pathways that extended from the mantle lithosphere across the Moho into the upper crust [97].

The results from this study, in combination with reassessment of previously published He-Ar isotopic data [54,90], provide clues related to the involvement of the ophiolitic fragments or oceanic relics in the formation or modification of auriferous ore fluids in the Ailaoshan belt. The varied ^3^He/^4^He ratios of 0.03–1.34 Ra and ^40^Ar/^36^Ar ratios of 310–4020 of the auriferous fluids indicate that they contain a component of altered oceanic crust (AOC) (Fig. 4d), in addition to a component from metasomatized mantle lithosphere, the lamprophyre source. Binary mixing modeling of ^3^He/^4^He and ^40^Ar/^36^Ar ratios suggests that the AOC components account for 30% (Fig. 4d). It is most likely that the AOC components were derived from serpentinized ultramafic rocks, altered oceanic relics, in the ophiolitic melanges that were underthrust northeastward along the steeply dipping ALF [33]. These serpentinized ultramafic rock blocks are interpreted to represent crustal fluid conduits, in which lithological heterogeneities were utilized by advecting ore fluids. The water/rock interaction during fluid migration in the middle-upper crust could have increased the fluid fertility.

### Lithospheric control over orogenic gold deposits and its implications

Our results show that the lithospheric architecture was essentially characterized by mechanical decoupling between crust and mantle, formed as a result of asthenosphere upwelling and lateral flow. This architecture promoted the partial melting of metasomatized mantle lithosphere and provided a favorable space for ponding and degassing of mantle-derived melts and a well-linked structural architecture for transport of melts and fluids, thus controlling the evolution of the Ailaoshan orogenic gold system.

Such a lithospheric structure, characterized by crust–mantle decoupling, is also interpreted for world-class giant gold provinces worldwide. The Jiaodong gold province was formed during the transition of subduction direction of the Pacific plate from NW to NNW at ca. 120 Ma [4,9]. The strike-slip movement along the Tanlu fault, a trans-lithospheric fault in the Jiaodong Peninsula, was NNE-trending, nearly perpendicular to the SKS fast direction in the mantle [98], thus showing the feature of crust–mantle decoupling (Fig. 6). Such a lithospheric structure promotes the degassing of the hydrous basic magmas at the base of the crust or during ascent, as suggested by previous studies based on the close spatial-temporal association between the mafic dikes and gold deposits in the Jiaodong gold belt [12,99]. Similarly, in the Xiaoqinling Orogen, central China, the crust with E-W-trending movement overlaps the underlying upper mantle with an N-S-trending SKS fast direction [100]. Moreover, in South China, the NE-trending orogenic belt overlies a nearly N-S-trending mantle SKS fast direction [101]. The Juneau gold belt, Alaska, along the Pacific eastern subduction zone, is also a potential analog of the Ailaoshan and Jiaodong gold provinces. This gold belt formed at ∼55 Ma, when a change from orthogonal to oblique subduction caused a shift from thrusting to strike-slip motion on the NNW-trending ore-controlling Sumdum and Fanshaw faults [102]. All these examples indicate that the critical architecture characterized by crust–mantle decoupling is typically formed during a transition period between tectonic regimes, driven by change in drift direction of the subducting plate and the orientation of the crust in orogenic settings. The obvious discrepancy between the subduction direction and orientation of crustal deformation reflects a lithospheric architecture characterized by crust–mantle decoupling, in which the orogenic gold deposits discussed above formed.

The reason why crust–mantle decoupling is beneficial for gold mineralization is that it has the capacity to connect the following processes that occur at different depths in the crust–mantle system. They include: (i) a deep thermal engine represented by upwelling asthenosphere, which triggered partial melting of metasomatized mantle lithosphere to generate basic-ultrabasic magma; (ii) crust–mantle decoupling, which provided a dilatational space for ponding and degassing of the basic magma at the base of the crust; and (iii) crustal-scale strike-slip faults that serve as heat-flow conduits for magma intrusion and fluid migration. We suggest that crust–mantle decoupling provides an optimal configuration related to a transition in tectonic regime, coincident with the presence of trans-lithosphere faults, and mafic dike emplacement, that is critical to the formation of specific orogenic gold deposits. Such lithosphere architecture is normally developed at craton or thick lithosphere margins, which provide high-quality exploration targets for a variety of ore deposit types [103]. From another perspective, the metallogenic model proposed here implies that despite the fact that asthenosphere upwelling can occur in different tectonic regimes [103,104], it is only capable of triggering gold mineralization with the additional key parameters of mantle lithosphere compositions and geometries, trans-crustal and trans-lithosphere structures, and suitable trap rocks and structures. This metallogenic model provides a new lithospheric control and petrological and geochemical constraints on the subcrustal model for the formation of OGDs.

## CONCLUSIONS

The multidisciplinary research carried out in this study has reconstructed the architecture of the lithosphere on the southeastern margin of Tibet and deciphered its control over the formation of OGDs along the Ailaoshan gold belt. The area is characterized by a lithosphere that was shaped by the decoupled deformation of crust and mantle that was, in turn, driven by deep Indian continental subduction. This architecture facilitated the development of heat-flow conduits and exerted structural control over the generation of OGDs. The formation of OGDs was initiated by mantle partial melting, basic magma ponding and fluid release at the base of the crust, as well as fluid migration in the middle-upper crust with similar geophysical signatures to that of the supergiant Olympic Dam iron-oxide copper-gold deposit. Similar lithosphere architecture is recognized in some giant gold provinces, implying that analogous geodynamic processes controlled their formation.

## METHODS

To image the lithosphere structure for the Ailaoshan gold belt, we jointly used seismic and MT imaging methods. For seismic imaging, we adopted a multi-scale strategy to first image mantle structure for southwest China using the teleseismic double-difference tomography method [105]. We then produced a Vp/Vs model of the lithosphere and the velocity gradients across the Moho by joint inversion of seismic body wave arrival times, surface wave dispersion data and receiver functions [106]. The details of different inversions and results are given in the Supplementary Data.

For MT imaging, based on the previous 3D electrical resistivity model of the lithosphere in the area [33], we added two horizontal (at 24°N and 25°N) and longitudinal profiles (at 100°E and 101°E), with the total MT stations increasing from 173 to 309 (Supplementary Data). The electromagnetic inversion system ModEM [107,108] was used to invert the full impedance tensor with a homogeneous 100 Ωm half-space starting model. The final total RMS (root mean square) misfit value reached 2.20 after 160 iterations (Supplementary Data), and a new more refined resistivity model with a wider area than the previous model [33] was obtained (Figs 3, S3). The details of the inversion and the model are presented in the Supplementary Data file.

The ∼70-mg-sized aliquots of high-purity separates of gold-related quartz that were irradiated in the McClellan Research Reactor Center at UC Davis were analyzed by crushing steps *under a vacuo condition*. A MAP215-50 noble gas mass spectrometer was used to expand the purified noble gases for Ar isotope analysis, with the data reduction based on ref. [109]. The 1 sd uncertainties for Br/Cl and I/Cl are ∼3% and ∼5%, respectively [110]. Helium and Ar isotopes of ore pyrites were measured at the Institute of Geology and Geophysics, Chinese Academy of Sciences, Beijing, using the Helix SFT mass spectrometer. The operating procedures and sample measurements data can be found in refs [111,112].

Electron probe microanalysis (EPMA), for major-element analysis of phlogopite, was carried out at the Geological Analysis Unit (GAU), Macquarie University, using a Cameca SX-100 electron microprobe with five wavelength-dispersive spectrometers. Minerals were analyzed using a 15 kV accelerating voltage, with a beam current of 20 nA and diameter of 1–2 μm. Counting times were 10 s for peak and 5 s for background measurements on each side of the peak. Natural minerals and synthetic oxides were used as standards for correction [119]. The temperature and pressure calculation for phlogopite are given in the Supplementary Data.

## Supplementary Material

nwac257_Supplemental_Files
