# Supplementary material for: Lithosphere architecture characterized by crust–mantle decoupling controls the formation of orogenic gold deposits
Source: Natl Sci Rev. 2022 Nov 16;10(3):nwac257. doi: 10.1093/nsr/nwac257 (PMC9985176; doi:10.1093/nsr/nwac257)
Supplement: nwac257_Supplemental_Files [file nwac257_supplemental_files.zip › Supplementary_Materials_and_Figures—HZ-w-1014.doc]

**Lithosphere architecture characterized by crust-mantle decoupling controls the formation of orogenic gold deposits**

（Supplementary Figures）

**Fig. S1** Cross sections of the Vp and Vs models along profiles at latitude (a) 23, (b) 24, and (c) 25N. Dashed lines indicate Moho interfaces determined by Ref [1].

**Fig. S2** Cross sections of Vp/Vs models along profiles at latitude (a) 23, (b) 24, and (c) 25N. Dashed lines indicate Moho interfaces determined by Ref [1].

**Fig. S3** Three-dimensional resistivity model projected on the five profiles. The white dashed lines denote the Moho [2], the black dashed lines denote the LAB [3]. The red stars represent the locations of ore deposits in Ailaoshan.

**Lithosphere architecture characterized by crust-mantle decoupling controls the formation of orogenic gold deposits**

（Supplementary Materials）

**1. Methods for seismic imaging**

**1.1 Improving the velocity models of the lithosphere of southeast margin of Tibetan plateau from joint inversion of surface wave, body wave and receiver functions**

To better determine the velocity models of the lithosphere beneath the southeastern margin of the Tibetan plateau, we have adopted the joint inversion method that incorporates body wave travel times, surface wave dispersion curves and receiver functions developed by Ref [4]. Due to complementary sensitivities of the three types of data, the joint inversion algorithm has the ability to better determine the velocity models of the lithosphere, especially the velocity variations across the Moho. The joint inversion algorithm is built upon the surface wave and body wave joint inversion of Ref [5] by incorporating receiver functions in the inversion system, following the algorithm proposed by Ref [6] to linearize the inversion of RFs. For details on the method, please refer to Ref [4].

Here we use the same surface wave dispersion data and body wave arrival time data of Ref [7], and the receiver functions data of Ref [8] for conducting the joint inversion (Supplementary Figures 1-3). The joint inversion grid has an interval of 0.5 degrees in the horizontal plane and has the interval of 5 km in the depth above 50 km and 10 km below 50 km. As shown in Ref [7], the current surface wave dispersion data and body wave arrival time data have the ability to resolve the model discretized above. By incorporating receiver functions, velocity models can be further improved [4]. The checkerboard resolution test shows that both Vp and Vs models are well resolved at different depth slices in the study region (Supplementary Figures 4 and 5). After inversion, the RMS data residuals decrease from 0.95 s to 0.43 s for body wave arrival times, from 3.6 s to 2.1 s for surface wave travel times at different periods, and from 0.031 1/s to 0.021 1/s for receiver functions. Compared to the model of Ref [7] from joint inversion of surface wave and body wave data, the new joint inversion model by also including receiver functions can better fits the receiver functions (Supplementary Figure 6), indicting the velocity variations across the Moho is better resolved. We also show the comparison of Vs models along two profiles at latitude of 29 and 31 degrees from different joint inversions (Supplementary Figure 7). The new joint inversion model by including the receiver functions is more compatible with the Moho interface. And across the Moho interface, the velocity discontinuities are more evident, suggesting the velocity variations across the Moho are better resolved. From the inverted Vs model, we can derive the Vs gradients across the Moho.

It is known that Vp/Vs ratios are more sensitive to the existence of fluids and melts than individual Vp and Vs models. Because of comparable resolutions between Vp and Vs models (Supplementary Figs. 4 and 5), we have determined Vp/Vs models by directly dividing Vp and Vs models.

**Supplementary Figure 1** Dataset of body wave arrival times used in this study, (a) Distribution of seismic events (dots) and stations (triangles). (b) P (red) and S (blue) travel time curves for events and stations in (a).

**Supplementary Figure 2** Dataset of surface wave dispersion curves used in this study. (a) Distribution of seismic stations (triangles) used for constructing two-station surface waves from ambient seismic noise. Different colors represent different sets of stations. (b) Rayleigh wave dispersion curves used in this study.

**Supplementary Figure 3** Dataset of receiver functions used in this study. (a) Distribution of teleseismic events used for constructing receiver functions. (b) Distribution of seismic stations on which receiver functions are available. (c) Distribution of interpolated receiver functions.

**Supplementary Figure 4** Recovered checkerboard Vp model at different depths for the study region from joint inversion of surface wave, body wave and receiver functions.

**Supplementary Figure 5.** Recovered checkerboard Vs model at different depths for the study region from joint inversion of surface wave, body wave and receiver functions.

**Supplementary Figure 6.** Comparison of observed receiver functions and predicted receiver functions based on the joint inversion Vs model of surface and body wave data (blue line), and the joint inversion Vs model of surface wave, body wave and receiver functions data (red line) at three positions, respectively.

**Supplementary Figure 7** Comparison of Vs models along latitudes of 31 and 29 from different joint inversions. (**a**) Initial Vs model; (**b**) Vs model from joint inversion of surface wave and body wave data. (**c**) Vs model from joint inversion of surface wave, body wave and receiver functions. Dashed lines indicate Moho interfaces determined by Ref [1]. ANHF=Anninghe Fault, LMSF=Longmenshan Fault; XSHF=Xiangshuihe Fault

2. **Methods for MT study of lithosphere structure**

To image the lithosphere structure of the Ailaoshan gold system, we carried out an extended study based on the 3D magnetotelluric (MT) survey in the RRF zone and adjacent areas [9]. Two horizontal profiles in the north (L1 & L2 in Supplementary Figure 8) and two longitudinal profiles in the west (P1 & P2 in Supplementary Figure 8) were added respectively, and the total MT stations increase from 173 to 309 (Supplementary Figure 8). In order to facilitate the comparison with the previous study [9], the inversion parameters of this study are consistent with pervious. The data in this survey were collected in 2016-2020 and include both broadband MT (BBMT) and long-period MT (LMT) data. The 309 BBMT stations (Supplementary Figure 9, blue dots) were recorded using Phoenix Geophysics MTU-5A instruments, and LMT data were collected at 88of the BBMT stations using LEMI-417 instruments (Supplementary Figure 9, red triangles). The recording time at the BBMT stations was 20-48 hours resulting in impedance data in the period band of 0.003–1,000 s. At each LMT station data were recorded for 10-15 days resulting in impedance data in the period band of 10-10,000 s. BBMT time series data were processed with the statistically robust algorithm of Ref [10] and Phoenix Geophysics SSMT2000 software, and LMT data were processed with the PRC-MTMV software [11]. A period band of 0.003–10,000 s was obtained by combining the broadband and long-period data.

The phase tensor approach of Ref [12] was used to inspect measured MT data, which is a widely used method for understanding the dimensionality of MT data. The MT data phase tensors are shown in Supplementary Figure 9a-c. At short period (T=0.24s), alignment and high ellipticities of the phase tensor principal axes coincide with mapped faults and geological boundaries such as the RRF. The increase in skew value in most phase tensor ellipses at periods around 127 s reflects the strong lateral heterogeneities of the resistivity structure in the mid-lower crust, and most skew values are greater than 3˚. At long period (T=1190s) the signals are sampling the upper mantle, and the phase tensors appear to show evidence for a 3D resistivity structure at this depth. In summary, the 3D characteristics in medium and long periods are very obvious, indicating the importance of 3D in version of the study area.

The Modular Electromagnetic Inversion system (ModEM) [13,14] was used for the 3D inversion of the Ailaoshan gold system data. The full impedance tensor was inverted, and only the high-quality BBMT data in the period band of 0.01-1,000 s and LMT data in the period band of 10-10,000 s were used. Error floors of 10% of |*Z*_xy_·*Z*_yx_|^1/2^ were assigned to all four impedance elements, where *Z* is the complex impedance. Data consisted of 309 stations and 27 periods. The central region of the study area was 660 x 360 km in extent and was discretized into horizontal cells that were 5 x 5 km. The grid was padded with 16 cells whose size grew with a geometric factor of 1.3 to cover an area of 1765 km × 1610 km. In the vertical direction the cells began with a thickness of 50 m and then increased with a geometric factor of 1.1 to a maximum depth of 1024 km. The grid had 108 × 170 × 80 cells in the x, y, and z directions respectively. The covariance parameters αx, αy, and αz were set to the same values (0.3). The inversion began from a homogeneous 100 Ωm half-space starting model. The final total r.m.s. misfit value of 2.20 was obtained after 160 iterations (Supplementary Figs. 8 and 9).

Because the resistivity model mostly focuses on the relationship between the deep electrical structure and the four typical deposits (Laowangzhai, Mojiang, Habo and Daping; green circles in Supplementary Figure 9) of the Ailaoshan gold system, we plotted three horizontal profiles (L2, Figure 3c; L3 & L4, Supplementary Figure 9 (a, b) and three longitudinal profiles (P3, P4, P5, Supplementary Figure 9 (c-e) which close to the deposits, to have high-resolution image up to a depth of 200 km. The main anomalies in the southern part of the new model are basically the same as the previous results, the electrical structure is further refined, especially the deep heat-flow conduits of ore-forming minerals are better described.

**Supplementary Figure 8** Topographic map of southeastern margin of Tibet showing location of the study region (white box). Blue dots represent the BBMT stations, and red triangles represent the LMT stations, while two symbols at the same point indicate that the BBMT and LMT were collected at the same station. Light blue lines represent the profiles used in the discussion. Black lines represent faults [9]. Abbreviation: tectonic units: ICB, Indo–China Block; IP, Indian Plate; SCB, South China Block; SCS, South China Sea; SYDB, Sichuan–Yunnan Diamond Block; TP, Tibetan Plateau; and faults: CTF, Chuxiong–Tonghai Fault; FNF, Funing–Napo Fault; LZJF, Luzhijiang Fault; LCJF, Lancangjiang Fault; MLF, Mile Fault; NTHF, Nantinghe Fault; RLF, Ruili–Longling Fault; RRF, Red River Fault; WLSF, Wuliangshan Fault; WMF, Wenshan–Maliupo Fault; XJF, Xiaojiang Fault; YMF, Yuanmou Fault.

**Supplementary Figure 9** (a-c) Phase tensor map at periods of 0.24s, 127s, and 1010s. (d) Average r.m.s. misfit map of each MT station over the period range 0.01-10,000 s from 3D inversion. Black solid lines denote normal and strike slip faults.

We have calculated the resolution in the vertical direction and that in the horizontal direction and confirmed the reliability of conductors below 40 km.

(1) **The resolution ratio of MT**

The resolution of the MT method includes the resolution in the vertical direction and that in the horizontal direction. The resolution in the vertical direction mainly depends on the wavelength of the electromagnetic wave, so it is often related to the observation frequency and the subsurface resistivity value. In general, the higher the observation frequency and the smaller the background resistivity, the higher the resolution in the vertical direction, while the high frequency corresponds to the shallow part, so the resolution of the shallow part is usually higher and the resolution of the deep part is lower. The resolution in the horizontal direction depends on the site spacing, the smaller the site spacing, the higher the resolution in the horizontal direction.

For the Ailaoshan gold belt, the background resistivity is generally low, and the average resistivity can be considered as 100 Ωm. The lowest frequency of broadband MT and long-period MT can reach 3000 s and 10000 s, respectively. According to the skin depth formula, even if only broadband data is considered, the detection depth is more than 200 km, thus the detection depth of 160 km is credible (Supplementary Figure 10).

**(2)** **Sensitivity test for conductors under 40 km**

In order to further confirm the reliability of conductors below 40 km, a group of sensitivity tests have been conducted. Firstly, the resistivity less than 50 Ωm was increased to 300 Ωm at the depth range 40-160 km (Supplementary Figure 10). Secondly, forward modeling was then used to calculate the response of the modified model. Lastly, calculate the change in the r.m.s. misfit between the modified model and the original model (Supplementary Figure 11). The change in r.m.s. misfit is more significant for the long periods that are sensitive to the investigation depth below 500 s, and less obvious at shorter periods at 45 s. The sensitivity test indicated that the MT data in the study is sensitive to deep features. Therefore, the high conductivity anomalies deeper than 40 km are real and credible.

**Supplementary Figure 10** The sensitivity test for conductors under 40 km (24° N). The gray regions are the ranges where the low resistivity was replaced by high resistivity.

**Supplementary Figure 11** The change in the r.m.s. misfit between the modified model compared to the original inversion model. The gray regions are the ranges where the low resistivity was replaced by high resistivity.

**3. Methods for temperature and pressure calculation of phlogopite in lamprophyre**

The temperature and pressure calculation of phlogopite as follows:

(1) T=a_1_/(lnD^TiO2^- b_1_)
(2) P=T× [(lnD^BaO^ - a_2_ - b_2_/T-d*lna_H2O_)/c]

a_1_=17600, b_1_ = -12.1; D^TiO2^ = TiO_2_ wt% in phlogopite/TiO_2_ wt% in glass; a_2_ = -2.167, b_2_ = 4553, c=-130.7, d=-0.388; D^BaO^ = BaO wt% in phlogopite/BaO wt% in glass; We have set a_H2O_ = 1.T is in Kelvins, P is in kbar [15,16].

**4 Lead isotopic compositions for ore sulfides and lamprophyre veins**

**Supplementary Figure 12** (a) ^207^Pb/^204^Pb vs. ^206^Pb/^204^Pb and (b) ^208^Pb/^204^Pb vs. ^206^Pb/^204^Pb diagram for the gold deposits and lamprophyres in the Ailaoshan orogenic gold belt. Data sources: Refs [17–23].

**References:**

1. Cheng J, Zhang J, Zhao M *et al.* Spatial distribution and origin of the high-velocity lower crust in the northeastern South China Sea. *Tectonophysics* 2021; **819**: 229086.

2. Wu T, Zhang S, Li M *et al.* Two crustal flowing channels and volcanic magma migration underneath the SE margin of the Tibetan Plateau as revealed by surface wave tomography. *Journal of Asian Earth Sciences* 2016; **132**: 25–39.

3. Hu J, Yang H, Li G *et al.* Seismic upper mantle discontinuities beneath Southeast Tibet and geodynamic implications. *Gondwana Research* 2015; **28**: 1032–47.

4. Han S, Zhang H, Gao L *et al.* Joint inversion of body wave arrival times, surface wave dispersion data and receiver functions: Method and Application to south China. *Journal of Geophysical Research: Solid Earth*:in press.

5. Han S, Zhang H, Xin H *et al.* USTClitho2.0: Updated Unified Seismic Tomography Models for Continental China Lithosphere from Joint Inversion of Body-Wave Arrival Times and Surface-Wave Dispersion Data. *Seismological Research Letters* 2022; **93**: 201–15.

6. Julià J, Ammon CJ, Herrmann RB *et al.* Joint inversion of receiver function and surface wave dispersion observations. *Geophysical Journal International* 2000; **143**: 99–112.

7. Liu Y, Yao H, Zhang H *et al.* The Community Velocity Model V. 1.0 of Southwest China, Constructed from Joint Body-and Surface-Wave Travel-Time Tomography. *Seismological Society of America* 2021; **92**: 2972–87.

8. He R, Shang X, Yu C *et al.* A unified map of Moho depth and Vp/Vs ratio of continental China by receiver function analysis. *Geophysical Journal International* 2014; **199**: 1910–8.

9. Yu N, Unsworth M, Wang X *et al.* New Insights Into Crustal and Mantle Flow Beneath the Red River Fault Zone and Adjacent Areas on the Southern Margin of the Tibetan Plateau Revealed by a 3‐D Magnetotelluric Study. *J Geophys Res Solid Earth* 2020; **125**, DOI: 10.1029/2020JB019396.

10. Egbert GD. Robust multiple-station magnetotelluric data processing. *Geophysical Journal International* 1997; **130**: 475–96.

11. Varentsov IM, Sokolova EY, Martanus E *et al.* System of electromagnetic field transfer operators for the BEAR array of simultaneous soundings: methods and results. *Izvestiya Physics of the Solid Earth* 2003; **39**: 118–48.

12. Caldwell TG, Bibby HM, Brown C. The magnetotelluric phase tensor. *Geophysical Journal International* 2004; **158**: 457–69.

13. Egbert GD, Kelbert A. Computational recipes for electromagnetic inverse problems: Computational recipes for EM inverse problems. *Geophysical Journal International* 2012; **189**: 251–67.

14. Kelbert A, Meqbel N, Egbert GD *et al.* ModEM: A modular system for inversion of electromagnetic geophysical data. *Computers & Geosciences* 2014; **66**: 40–53.

15. Guo J, Green TH. Experimental study of barium partitioning between phlogopite and silicate liquid at upper-mantle pressure and temperature. *Lithos* 1990; **24**: 83–95.

16. Righter K, Carmichael ISE. Phase equilibria of phlogopite lamprophyres from western Mexico: biotite-liquid equilibria and P - T estimates for biotite-bearing igneous rocks. *Contributions to Mineralogy and Petrology* 1996; **123**: 1–21.

17. Chen Y, Liu J, TRAN My D *et al.* Regional Metallogenesis of the Chang’an Gold Ore Deposit in Western Yunnan: Evidences from Fluid Inclusions and Stable Isotopes. *Acta Geologica Sinica - English Edition* 2010; **84**: 1401–14.

18. Lu Y-J, Campbell McCuaig T, Li Z-X *et al.* Paleogene post-collisional lamprophyres in western Yunnan, western Yangtze Craton: Mantle source and tectonic implications. *Lithos* 2015; **233**: 139–61.

19. Yuan S, Ge L, Guo X *et al.* Lead and sulfur isotope characteristics and geological implication of Daping gold deposit. *Journal of Guilin University of Technology* 2011; **31**: 11–8 (in Chinese with English abstract).

20. Zhang J, Wu J, Li C *et al.* Geological features and genesis of Laowangzhai gold deposit in Zhenyuan County, Yunnan. Gold 2010; **31**: 19–23 (in Chinese with English abstract).

21. Li S, Zhang J, Yang L *et al.* Origin of metallogenic materials of the Chang’an gold deposit in the southern Ailaoshan belt: evidence from sulfur and lead isotopic composition. *Geoscience* 2013; **27**: 879–87 (in Chinese with English abstract).

22. Xu X-W, Zhang B-L, Qin K-Z *et al.* Origin of lamprophyres by the mixing of basic and alkaline melts in magma chamber in Beiya area, western Yunnan, China. *Lithos* 2007; **99**: 339–62.

23. Ying H, Liu H, Yand X *et al.* Geology and origin of Tongchang gold deposit in Yunnan province. *Geology and Resources* 2006; **15**: 265–71 (in Chinese with English abstract).
